# Advanced Modulation Formats for 400 Gbps Optical Networks and AI-Based Format Recognition

**DOI:** 10.3390/s24227291

**Published:** 2024-11-14

**Authors:** Zhou He, Hao Huang, Fanjian Hu, Jiawei Gong, Binghua Shi, Jia Guo, Xiaoran Peng

**Affiliations:** 1 Hubei Key Laboratory of Digital Finance Innovation, Hubei University of Economics, Wuhan 430205, China; hezhou@hbue.edu.cn; 2 School of Information Engineering, Hubei University of Economics, Wuhan 430205, China; 3 Wuhan 2nd Ship Design and Research Institute, Wuhan 430205, China; 4 School of Mechanical and Electrical Engineering, Hubei University of Education, Wuhan 430205, China; 5 Faculty of Computer and Information Sciences, Hosei University, Tokyo 102-8160, Japan; 6 Linktel Technologies Co., Ltd., Wuhan 430072, China

**Keywords:** modulation format, optical networks, convolutional neural network (CNN), modulation format identification (MFI), integration of communication and sensing (ICAS)

## Abstract

The integration of communication and sensing (ICAS) in optical networks is an inevitable trend in building intelligent, multi-scenario, application-converged communication systems. However, due to the impact of nonlinear effects, co-fiber transmission of sensing signals and communication signals can cause interference to the communication signals, leading to an increased bit error rate (BER). This paper proposes a noncoherent solution based on the alternate polarization chirped return-to-zero frequency shift keying (Apol-CRZ-FSK) modulation format to realize a 4 × 100 Gbps dense wavelength division multiplexing (DWDM) optical network. Simulation results show that compared to traditional modulation formats, such as chirped return-to-zero frequency shift keying (CRZ-FSK) and differential quadrature phase shift keying (DQPSK), this solution demonstrates superior resistance to nonlinear effects, enabling longer transmission distances and better transmission performance. Moreover, to meet the transmission requirements and signal sensing and recognition needs in future optical networks, this study employs the Inception-ResNet-v2 convolutional neural network model to identify three modulation formats. Compared with six deep learning methods including AlexNet, ResNet50, GoogleNet, SqueezeNet, Inception-v4, and Xception, it achieves the highest performance. This research provides a low-cost, low-complexity, and high-performance solution for signal transmission and signal recognition in high-speed optical networks designed for integrated communication and sensing.

## 1. Introduction

The pervasive intelligence of future 6G networks will enable wireless networks to accurately sense the environment, providing massive and reliable data for real-time decision-making by network artificial intelligence. This organic empowerment of communication and sensing will be one of the core functionalities of 6G technology [1].

In recent years, the continuous development and maturation of optical fiber sensing technology have endowed fiber-based transmission networks with the potential for the seamless integration of communication and sensing functions. Traditional sensing systems based on local computing and independent perception are gradually becoming inadequate to support the extreme sensing demands of various new applications. Optical communication networks, with their high-bandwidth, long-distance, low-latency, and highly reliable data transmission capabilities, can effectively assist in achieving collaborative sensing across multiple nodes, thus broadening the scope of perception. These networks also serve as the foundation for computational infrastructure, allowing massive amounts of sensing data to be transmitted to widely distributed multi-level computational nodes via optical communication networks. Combined with technologies such as artificial intelligence, this enables customized feature extraction, deep computation, intelligent recognition, and information fusion, forming a highly efficient and mutually enhancing architecture system characterized by high-bandwidth, low-latency communication; real-time status sensing; and on-demand computational resource scheduling [2].

Currently, the application scenarios of optical network ICAS technology mainly include the operation and maintenance of optical fiber resources, smart cities, and pipeline and structural monitoring, as well as geological and environmental monitoring.

### 1.1. Prior Works for Optical Modulation Technologies

Advanced optical modulation formats are crucial to the development of high-speed optical networks. They can meet the continuously growing demand for system data throughput and improve spectrum efficiency. However, multi-level modulation not only requires a higher optical signal-to-noise ratio (OSNR) but is also more sensitive to fiber nonlinear effects and laser phase noise, both of which limit the maximum transmission distance of optical networks. To date, there has been much new research progress in the modulation format field of high-speed optical communication systems in the industry [3,4,5,6]. Studies have shown that 50 Gb/s alternate polarization frequency shift keying (Apol-FSK) signals exhibit better transmission performance than carrier-suppressed return-to-zero frequency shift keying (CSRZ-FSK) and return-to-zero differential phase shift keying (RZ-DPSK) signals, making them one of the most promising solutions for future high-speed transmission systems [3]. A transceiver system using radio over fiber-based polarization division multiplexing differential quadrature phase shift keying (RoF-based PDM-DQPSK) DWDM was developed, and simulation results indicated that a maximum data rate of 1.792 Tbps can be achieved using 64-channel DWDM technology, with a maximum transmission distance extendable to 1600 km at a bit error rate (BER) of 10^−12^ [4]. An all-optical wavelength and format conversion scheme based on a delay line interferometer (DLI) and a pump-assisted nonlinear optical loop mirror (NOLM) can convert a 20 Gbps differential quadrature phase shift keying (DQPSK) signal to a four-level pulse amplitude modulation (PAM4) signal. This scheme can be deployed in all-optical gateways to bridge long-distance and short-distance optical networks [5]. A 50 Gbps quadrature phase shift keying (QPSK) and a 100 Gbps sixteen quadrature amplitude modulation (16QAM) signal have been demonstrated through all-optical conversion while maintaining an error vector magnitude (EVM) below 10% with a required bandwidth of 55 GHz. The results showed that the converted QPSK and 16QAM signals can be transmitted to 19,500 km and 4000 km, respectively, in single-channel and eight-channel transmission by maintaining a sufficient 62.5 GHz channel spacing [6]. Currently, more and more research efforts are focused on modulation format conversion, and there is still room for further exploration in the research of advanced modulation formats with high nonlinear resistance. FSK modulates signals by different frequencies with constant optical power, inherently exhibiting high nonlinear resistance, and has received more attention from researchers in recent years [7,8,9]. By integrating technologies such as FSK, alternating polarization, and CRZ, solutions can be provided for the development of high-speed computing optical networks.

This paper proposes a noncoherent solution based on the Apol-CRZ-FSK modulation format for achieving a 4 × 100 Gbps DWDM optical network. The narrow channel spacing in a DWDM system exacerbates nonlinear effects, necessitating a modulation format with high nonlinear tolerance to enhance system performance. To address this challenge, we conducted comprehensive research on Apol-CRZ-FSK in a DWDM context and compared to traditional CRZ-FSK and DQPSK modulation formats, Apol-CRZ-FSK exhibits superior nonlinearity resistance, supporting longer transmission distances and better transmission performance. Specifically, when the input optical power is low and nonlinear effects are negligible, the performance advantage of the Apol-CRZ-FSK modulation format in a 4 × 100 Gbps DWDM system is not significant. However, as the optical power increases and the transmission distance extends, the Apol-CRZ-FSK signal demonstrates better nonlinearity resistance, especially when nonlinear effects become the primary factor affecting system performance.

Despite its excellent nonlinearity resistance and transmission performance, the Apol-CRZ-FSK modulation format faces some potential limitations and challenges in practical applications. First, the generation and processing of Apol-CRZ-FSK signals require more complex equipment and technical support, which may increase the cost and complexity of the system. Second, in real-world network environments, nonlinear effects may become more severe due to factors such as fiber aging and temperature variations, thereby impacting the quality of signal transmission. Additionally, although the demodulation process of Apol-CRZ-FSK signals does not require complex coherent demodulation, direct detection methods may not achieve optimal performance in certain scenarios. Therefore, optimizing the generation and demodulation techniques of the Apol-CRZ-FSK modulation format to further enhance its performance in practical applications remains a critical direction for future research.

### 1.2. Prior Works for MFI

Telecommunications operators in various countries generally adopt different equipment of various speed levels from different manufacturers to construct the backbone network, metro network, and access network of computing optical networks, and carry service signals for different customers through different optical modulation technologies. Computing optical networks are evolving into more complex, dynamic, flexible, and intelligent systems. To meet the demand for signal transmission and various computing capabilities in the era of intelligent connection of everything, computing optical networks also need to perceive business and computing information; intelligently identify different modulation formats carrying signals from different customers; and dynamically adjust data transmission wavelength, signal modulation format, signal power, and other parameters based on link conditions and user service requirements. Therefore, ascertaining how to enhance the transmission performance of signals through advanced modulation formats and quickly distinguish signal formats in computing infrastructure to achieve intelligent modulation format identification (MFI) have become research hotspots.

Classical MFI algorithms are mainly divided into either likelihood ratio decision theory-based or feature extraction-based. Methods based on likelihood ratio decision theory require consideration of a large amount of prior knowledge such as the mean, variance, and SNR of communication signals [10]. This approach is computationally complex and lacks robustness. Methods based on feature extraction require less prior knowledge, are simpler to analyze, and have higher efficiency [11]. These methods distinguish between different modulation formats by extracting characteristic parameters from the signals [12,13] and are greatly influenced by the selection of features, and different features can lead to significant differences in recognition results. Therefore, determining how to select features is crucial to the quality of recognition results. Since feature extraction takes a certain amount of time, it is not possible to achieve real-time processing in practical applications.

In recent years, neural network technology has been widely used in real-time data processing, feature extraction, and image recognition. Research has shown that the recognition of 11 modulation types based on a time-frequency feature fusion strategy using a Squeeze-and-Excitation Network (SENet) achieved a maximum recognition accuracy of 92.5%, and the average recognition accuracy reached 90.87% above 0 dB, outperforming other deep learning algorithms [14]. The use of a joint DNN to recognize the amplitude statistical histogram of radio frequency signals achieved a recognition accuracy of 96.05%, significantly improving the recognition accuracy of radio frequency signals under low SNR conditions [15]. A scheme based on convolutional neural networks and equal weight multitask learning extracts shared features of the OSNR and modulation formats (10 Gbps QPSK, QAM8, QAM16, QAM64) from constellation diagrams and uses them for multi-label classification. Simulation results show that the accuracy of the MFI and OSNR can stably and continuously reach 100% [16].

Neural network technology utilizes deep neural networks with multiple nonlinear layers to learn and extract feature information from signals, without relying on manual experience to extract signal features [17]. MFI methods based on neural network technology have the advantages of requiring less prior knowledge, strong feature extraction capabilities, and high recognition accuracy, which have received widespread attention and research from scientific researchers [18,19,20].

### 1.3. Contribution

In the 400 Gbps high-speed optical network, nonlinear effects such as four-wave mixing and cross-phase modulation will become one of the main factors limiting the system’s transmission performance. A solution based on a novel modulation format, Apol-CRZ-FSK, to implement a 4 × 100 Gbps optical communication system is proposed in this paper. This modulation format is based on a dual-light-source scheme, combining the advantages of FSK and Apol modulation; by adding specific chirp amounts to the rising and falling edges of the pulses through a phase modulator, the chirp sign is opposite to the chirp generated by the self-phase modulation effect (SPM) on the optical pulses, further enhancing the ability of the optical signal to resist nonlinear effects in the optical fiber. Compared with traditional 4 × 100 Gbps CRZ-FSK and DQPSK systems, it has better transmission performance and higher nonlinearity resistance. The solution for the 4 × 100 Gbps Apol-CRZ-FSK modulation signals with high nonlinearity resistance does not require the replacement of optical fiber lines during the upgrading and renovation of traditional networks. Only the optical transmitter module needs to be replaced, and the receiving side does not require complex coherent demodulation. Direct detection can be used to achieve cost reduction and green energy conservation. In addition, in response to the transmission requirements and the signal perception and recognition requirements in future computing optical networks, this study identifies three different modulation formats through the Inception-ResNet-v2 neural network model, which can provide effective solutions for high-speed computing optical networks in the 6G era.

## 2. Materials and Methods

### 2.1. System Structure and Setup

**Figure 1:**
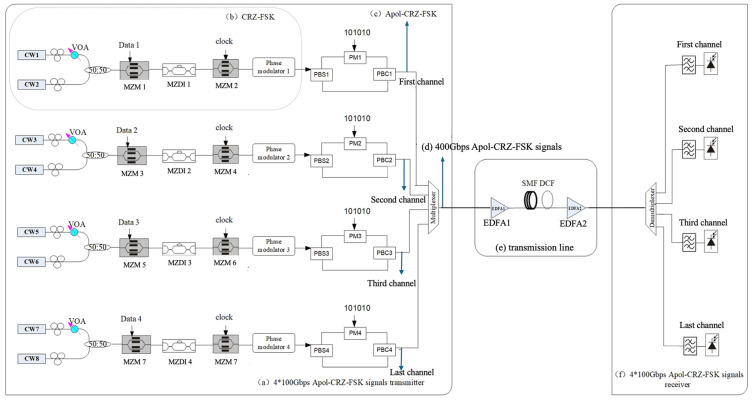
Architecture of a 4 × 100 Gbps Apol-CRZ-FSK signal transmission system for optical networks.

The structure of the 4 × 100 Gbps Apol-CRZ-FSK signal transmitters proposed in this study for optical networks is shown in Figure 1a. Each channel adopts a dual-light-source scheme, which involves two lasers generating two optical signals with the same intensity and a frequency spacing of 100 GHz to distinguish between digital signals 0 and 1. The two lasers for the first channel are denoted as CW1 and CW2, and similarly, the lasers for the second, third, and last channels are represented as CW3, CW4, CW5, CW6, CW7, and CW8, respectively.

Taking the modulation process of the first channel as an example, the two laser signals CW1 and CW2 are combined through a 3 dB coupler and then modulated by MZM1, Variable optical attenuator (VOA) is used to adjust the power level of the transmitter. First, the input 100 Gbps binary pseudo-random sequence data1 $\left\{b_{n}\right\}$ is differentially encoded to obtain a binary relative code sequence $\left\{d_{n}\right\}.$ The generation principle of the relative code sequence ${\;d}_{n}$ is as follows [3]:

(1) $$d_{n}=b_{n}⊕d_{n-1}$$

Here, ⊕ is known as the XOR gate in digital circuits.

The bias voltage of the MZM1 modulator is set at $V_{bias}=V_{\pi }$*,* and the input signal is the differentially encoded pseudo-random NRZ signal $V_{in}(t)=V_{\pi }a(t)$*,* $a\left(t\right)=\pm 1$. The phase difference between the code “0” and the code “1” of the signal is 180 degrees, suppressing the discrete components in the signal spectrum. The output modulated optical signal can be expressed as [3]:

(2) $$E_{out}\left(t\right)=j\cdot E_{in}(t)\cdot \sin\left[\frac{\pi }{2}a(t)\right]$$

(3) $$P_{out}\left(t\right)\;=\;\frac{P_{in}(t)}{2}\cdot \left\{1-\cos\left[\pi a(t)\right]\right\}=P_{in}(t)$$

Subsequently, the signal is demodulated in the MZDI 1 with a 1-bit delay. The output of the MZDI1 exhibits periodically varying comb-filter characteristics. When the CW1 and CW2 signals with a frequency spacing of 100 GHz are modulated to the peak and trough of the transmission curve of the MZDI1, respectively, the waveforms of the two frequencies outputted on the same interference arm are opposite, resulting in a phase difference of 180 degrees, which creates interference patterns of cancellation and enhancement between them.

This allows the data of CW1 and CW2 with different frequencies to be demodulated as intensity-modulated signals on “1” and “0”, respectively. The demodulation output of MZDI 1 exhibits complementary optical power on the two interference arms. Although these two demodulated signals are derived from the same source, they are logically opposite and complementary in power. In the time-domain optical intensity, they manifest as continuous light, while the optical frequency hops between two wavelengths as the information changes. Therefore, it can generate FSK signals that carry optical pulses in every bit interval.

MZM2 performs pulse slicing on the FSK signal with the bias point set at Vπ/2, and the amplitude and frequency of the clock signal are Vπ/4 and B, respectively. The phase difference between the two clock signals is π. A phase modulator1 is added afterwards to control the pre-chirp amount, which can generate the CRZ-FSK signal as shown in Figure 1b. If the CRZ-FSK signal is further processed through a polarization alternating device, an Apol-CRZ-FSK signal will be generated. The CRZ-FSK optical signal is divided into two optical signals with the same intensity and orthogonal polarization states by a 45-degree polarizing beam splitter (PBS). One of the signals is phase-modulated to vary its phase between 0 and π. The two polarization signals are then coupled by a polarizing beam combiner (PBC) to obtain the Apol-CRZ-FSK signal for channel 1 with orthogonal polarization states for adjacent bits, as shown in Figure 1c. Similarly, signals for the other three wavelength channels are generated. This project is implemented by VPI9.3 simulation.

**Figure 2:**
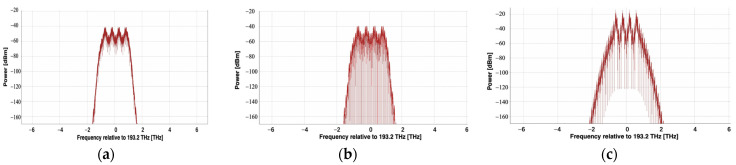
Spectral diagram of a 4 × 100 Gbps signals: (**a**) Apol-CRZ-FSK; (**b**) CRZ-FSK; (**c**) DQPSK.

The generated four 100 Gbps Apol-CRZ-FSK signals are coupled to a single optical fiber through a multiplexer, resulting in a 4 × 100 Gbps Apol-CRZ-FSK signal at the location shown in Figure 1d. Its spectral diagram is shown in Figure 2a. The single-span transmission line is shown in Figure 1e. Firstly, a 4 × 100 Gbps DWDM signal is power amplified through an EDFA1 amplifier with a noise figure of 4.5 dB. The dispersion-compensating fiber (DCF) on the line side, with a dispersion coefficient of −90 ps/nm/km, is used to compensate for signal distortion caused by the single-mode fiber (SMF) with a dispersion coefficient of 16 ps/nm/km. An EDFA2 amplifier on the receiving side of the signal is used for signal power amplification to compensate for signal attenuation during long-distance transmission before sending it to the receiver, as shown in Figure 1f. A demultiplexer divides the 4 × 100 Gbps signal into four 100 Gbps signals, which are then detected by an optical bandpass filter for frequency discrimination and directly received by the receiver.

### 2.2. Result Analysis

**Figure 3:**
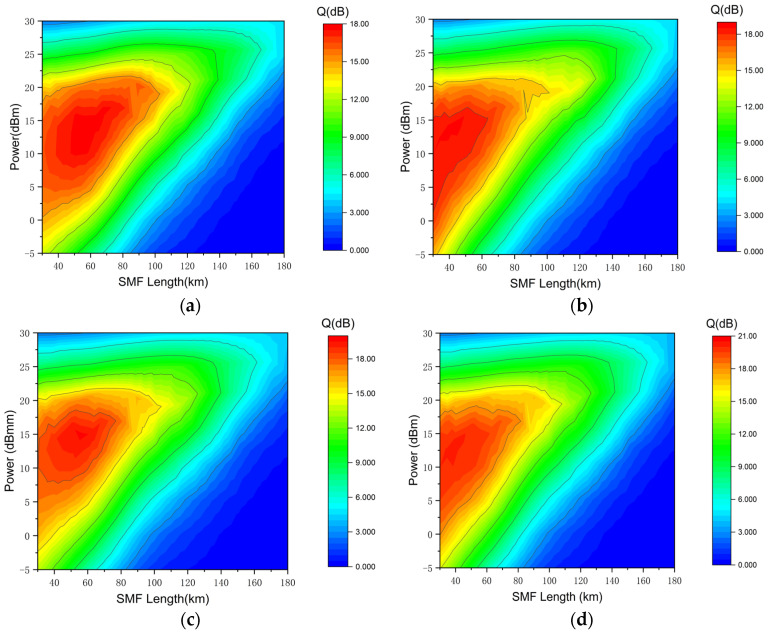
The relation among SMF length, Q-factor, and launch power for the four wavelength channels of 4 × 100 Gbps Apol-CRZ-FSK signal transmission: (**a**) first channel; (**b**) second channel; (**c**) third channel; (**d**) last channel.

In the 4 × 100 Gbps Apol-CRZ-FSK communication system, as the launch power varies from −5 dBm to 30 dBm, the OSNR of the signal increases with the increase in launch power, resulting in a gradual increase in the Q-factor of each channel signal. Figure 3 shows that the Q-factors of the four channels are 9.0 dB, 10.0 dB, 8.5 dB, and 10.0 dB, respectively, when the transmission distance is 120 km, and the launch power is 15 dBm. When the launch power is 20 dBm, the signal achieves optimal transmission performance, with Q-factors of 14.5 dB, 13.1 dB, 15.8 dB, and 14.4 dB for the four channels, respectively. As the launch power continues to increase, the impact of nonlinear effects such as four-wave mixing and cross-phase modulation caused by small-wavelength spacing and high-power density becomes increasingly severe, leading to continued degradation of the signal. When the launch power is 26 dBm, the maximum transmission distance of the 4 × 100 Gbps Apol-CRZ-FSK signals over a single span is 168 km.

To better assess the performance advantages and disadvantages, a comparative analysis was conducted among the 4 × 100 Gbps Apol-CRZ-FSK, CRZ-FSK and DQPSK systems under identical conditions. The single-channel 100 Gbps CRZ-FSK setup is depicted in Figure 1b, where signals from a four-channel 100 Gbps CRZ-FSK transmitter, employing a wavelength spacing of 100 GHz, are multiplexed and transmitted through a single fiber. The system configurations for the line side and receiver are identical to those used in the 4 × 100 Gbps Apol-CRZ-FSK, as illustrated in Figure 1e and Figure 1f, respectively. Figure 2b presents the optical spectrum of the 4 × 100 Gbps CRZ-FSK signals at the transmission side.

**Figure 4:**
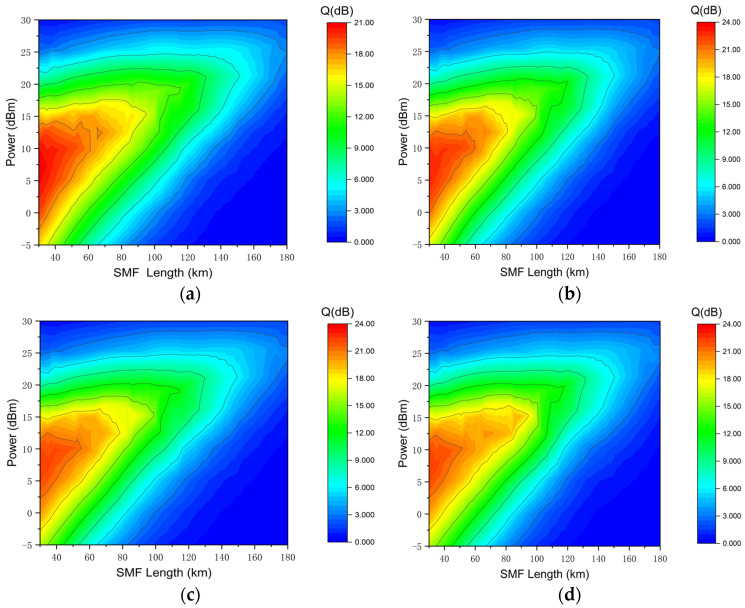
The relation among SMF length, Q-factor and launch power for the four wavelength channels of 4 × 100 Gbps CRZ-FSK signal transmission: (**a**) first channel; (**b**) second channel; (**c**) third channel; (**d**) last channel.

Examining the transmission performance of the 4 × 100 Gbps CRZ-FSK communication system in Figure 4, it is observed that at a transmission distance of 120 km and launch power of 15 dBm, the Q-factors of the four channels are 9.7 dB, 10.3 dB, 9.7 dB, and 9.5 dB, respectively. At this point, the difference in signal transmission performance between the CRZ-FSK and Apol-CRZ-FSK signals is minimal, approximately 1 dB. However, when the launch power is increased to 20 dBm, the Q-factors of the four channels of the 4 × 100 Gbps CRZ-FSK signals drop to 11.6 dB, 12.3 dB, 11.3 dB, and 11.9 dB, which are lower than those of the 4 × 100 Gbps Apol-CRZ-FSK signals by 2.9 dB, 0.8 dB, 4.5 dB, and 2.5 dB, respectively. Furthermore, at a launch power of 22 dBm, the maximum transmission distance over a single span is 152 km, a decrease of 16 km compared to the 4 × 100 Gbps Apol-CRZ-FSK signals.

For the 4 × 100 Gbps DQPSK system, a phase modulator and an MZM are used to generate each 100 Gbps DQPSK signal at the transmitting side. The modulation depth of the first phase modulator is π/2, and the modulation depth of the second MZM is π. The DQPSK modulation format has only half the symbol rate under the condition of a fixed transmission bit rate, which compresses the spectrum and allows for higher spectral efficiency in DWDM systems. It also improves resistance to dispersion. The signal from the four-channel 100 Gbps DQPSK transmitter with a wavelength spacing of 100 GHz is coupled through a wavelength multiplexer, and the resulting signal spectrum is depicted in Figure 2c. After long-distance transmission through the transmission line shown in Figure 1e, the 4 × 100 Gbps DQPSK signal is separated into four 100 Gbps signals by a demultiplexer at the receiving side. Each signal passes through an optical bandpass filter, is delayed by one symbol interval, and then multiplied by itself. After low-pass filtering and sampling decision, the original digital information can be directly recovered.

**Figure 5:**
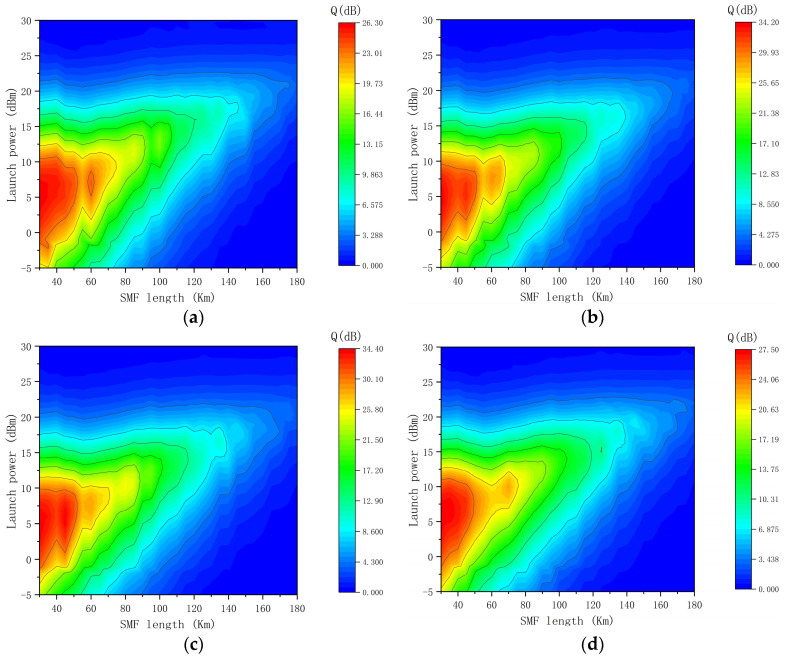
The relation among SMF length, Q-factor and launch power for the four wavelength channels of 4 × 100 Gbps DQPSK signal transmission: (**a**) first channel; (**b**) second channel; (**c**) third channel; (**d**) last channel.

Figure 5 illustrates the transmission performance of a 4 × 100 Gbps DQPSK signal. When the single span is 120 km and the launch power is 15 dBm, the 4 × 100 Gbps DQPSK signal achieves optimal transmission performance. At this point, the Q-factors for the four channels are 9.8 dB, 10.8 dB, 11.5 dB, and 9.9 dB, respectively. The performance difference between these channels and the four wavebands of Apol-CRZ-FSK is also minimal. However, when the launch power increases to 20 dBm, the Q-factors for the four signals drop to 5.7 dB, 6.2 dB, 6.4 dB, and 6.2 dB, respectively. These values are significantly lower than the Q-factors of the four signals of the 4 × 100 Gbps Apol-CRZ-FSK under the same conditions, with differences of 8.8 dB, 6.9 dB, 9.4 dB, and 8.2 dB, respectively. At a launch power of 19 dBm, the maximum transmission distance for a single span is 145 km, which is 23 km less than that of the 4 × 100 Gbps Apol-CRZ-FSK signals. Based on these data, it is evident that when the launch power is low and the impact of nonlinear effects is minimal, Apol-CRZ-FSK does not exhibit a significant performance advantage. However, as the optical power gradually increases and the transmission distance extends, nonlinear effects become the primary factor affecting system performance. In such cases, the Apol-CRZ-FSK signal demonstrates superior nonlinearity resistance, outperforming the other two signals in terms of both signal Q-factor and transmission distance.

**Figure 6:**
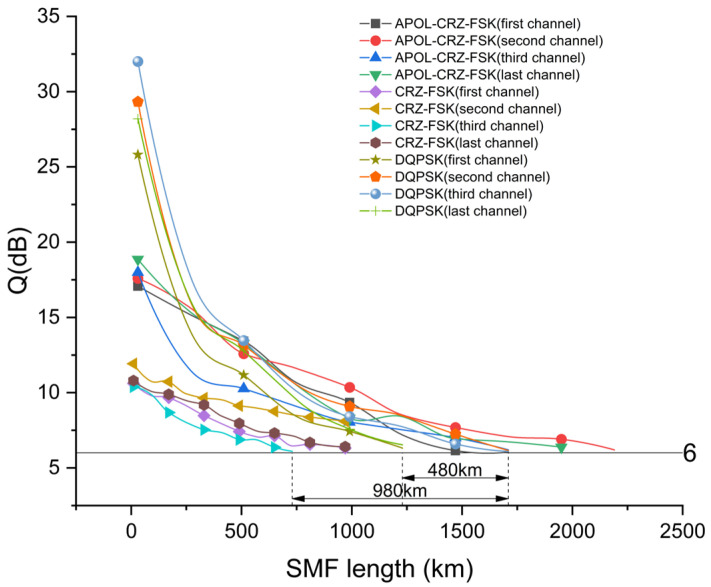
Performance analysis and comparison of three signals in different distances.

Based on the research of single-span transmission performance mentioned above, Figure 6 analyzes the transmission performance differences during multi-span transmission. In this scheme, each span is first passed through an EDFA amplifier to amplify the signal power before entering a single-mode fiber for transmission. Subsequently, dispersion compensation fibers of corresponding lengths are used for post-dispersion compensation. Due to the influence of factors such as four-wave mixing, cross-phase modulation, and dispersion in the 4 × 100 Gbps system, there are certain differences in the transmission performance of the four channels. The transmission distance of the channel with the worst performance should be selected as the transmission distance of the system. As can be seen from the figure, the maximum transmission distance of the 4 × 100 Gbps Apol-CRZ-FSK signal is 1740 km, at which point the Q-factors of the four channels are 6.1 dB, 7.0 dB, 6.3 dB, and 6.7 dB, respectively. The maximum transmission distance of the 4 × 100 Gbps DQPSK signal is 1260 km, which is 480 km shorter than the Apol-CRZ-FSK signal. The maximum transmission distance of the CRZ-FSK signal is 760 km, which is 980 km shorter than the Apol-CRZ-FSK signal.

**Figure 7:**
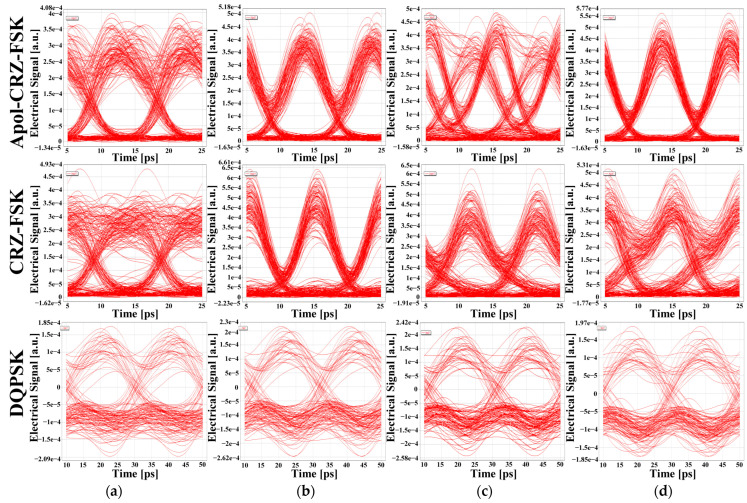
Eye diagrams of the four-channel signals for the three types of signals at the launch power of 6 dBm and transmission distance of 1500 km: (**a**) first channel; (**b**) second channel; (**c**) third channel; (**d**) last channel.

Figure 7 displays the eye diagrams of the first channel (Figure 7a), second channel (Figure 7b), third channel (Figure 7c), and last channel (Figure 7d) for each of the three signals when the launch power is 6 dBm and the signals are transmitted for 1500 km. At this point, the Apol-CRZ-FSK signal exhibits superior transmission performance.

## 3. AI-Based Modulation Format Recognition

### 3.1. Format Recognition System Based on Inception-ResNet-v2 Convolutional Neural Network Modeling

Service signals from different customer sides in the optical network may be carried through various modulation formats, requiring fast, flexible, and intelligent format recognition within the network. The recognition of three modulation formats is realized based on the Inception-ResNet-v2 convolutional neural network model in this study, and the core idea is to integrate the Inception module with the ResNet module, where the Inception module captures multi-scale features by parallelizing multiple convolutional kernels of different sizes, while the ResNet module addresses the issues of gradient vanishing and exploding in deep networks through residual connections, which facilitates the network’s learning of complex mapping relationships, thereby enhancing the model performance and stability, and facilitating better training of deep models [10,11]. Since the Inception-ResNet-v2 model can extract features from different angles and scales, it has a stronger capability to recognize subtle differences in optical communication signals. This study uses eye diagrams of signals to identify different modulation formats. When recognizing eye diagrams of different modulation formats, the Inception-ResNet-v2 model can capture unique features specific to each modulation format, thereby improving recognition accuracy.

**Figure 8:**
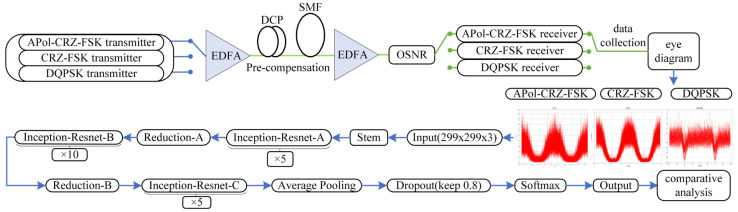
Model of the MFI method based on the Inception-ResNet-v2.

As shown in Figure 8, the signals emitted by the 100 Gbps Apol-CRZ-FSK, CRZ-FSK, and DQPSK signal transmitters studied in the previous text are transmitted through a single-mode fiber (SMF) with lengths ranging from 30 km to 180 km. Subsequently, post-dispersion compensation is performed using DCF of corresponding lengths. A set of OSNR components is used to load the defined noise floor into the signal, with the OSNR varying from 6 dB to 30 dB in steps of two. The eye diagrams of different modulation formats are captured on the receiving side of the system through the filter, and the processed data yield a 299 × 299 × 3 dataset for format recognition. Initially, the eye diagram data are input into a Stem layer, followed by passing through Inception-ResNet-A, Inception-ResNet-B, and Inception-ResNet-C sequentially, with a Reduction module inserted between each two layers. Then, the data are compressed and dimension-reduced through an Average Pooling layer to optimize and decrease network complexity. Finally, the data go through a Dropout layer and a Fully Connected layer, concluding with the output via a SoftMax function.

The Stem module in Figure 8 utilizes an adaptive deformable spatial transformer to reposition, scale, and rotate the input feature maps, endowing the network with robustness to different transformations of the input images, and automatically aligning features from various angles, sizes, and positions. Moreover, the Stem module adopts a branch-and-connect approach, merging the spatially transformed feature maps with the original ones, preserving the low-level features from the original feature maps while enhancing the ability to distinguish adversarial samples using the transformed feature maps. The 1 × 1 convolution operation primarily serves to reduce data dimensions and introduce nonlinearity, thereby improving the network’s expressive power. The Equation (4) for 1 × 1 convolution is as follows [21]:

(4) $${\;Conv}_{1\times 1}{\left(X\right)}_{i,j,k}=\sum_{c=1}^{C}W_{1,c,k}X_{i,j,c}+b_{k}$$

where $W_{1,c,k}$ represents the weight between the channel c and the channel *k* of the 1 × 1 convolution kernel, $b_{k}$ denotes the bias of the channel *k*, and ${\left(X\right)}_{i,j,k}$ signifies the value of the output feature map at the row *i*, column *j*, and channel *k*.

The Inception-ResNet-A module utilizes symmetric convolutional kernels, whereas the Inception-ResNet-B and Inception-ResNet-C modules employ asymmetric convolutional kernels such as 1 × 7, 7 × 1, 1 × 3, and 3 × 1 to reduce the computation time for parameter operations and increase the depth and nonlinearity of the network.

In this study, the Batch Normalization (BN) algorithm is used for normalization processing to prevent the activation function from entering the nonlinear saturation region, thereby avoiding the problem of gradient dispersion, and to enhance the network’s generalization capability. By introducing learnable reconstruction parameters *γ* and *β*, the network can learn to restore the original feature distribution of the network, as shown in Equation (5) [22].

(5) $$\;y^{\left(k\right)}=\gamma^{\left(k\right)}{\hat{x}}^{\left(k\right)}+\beta^{\left(k\right)}$$

When the condition in Equation (6) is met, the network can recover the original features learned by a specific layer [22]. $E\left[x^{\left(k\right)}\right]$ refers to the mean value of the neurons in a batch of data, and $Var(x^{\left(k\right)})$ refers to the standard deviation of the input values of the neurons in a batch of training data.

(6) $${\;\beta }^{\left(k\right)}=E\left[x^{\left(k\right)}\right],\gamma^{\left(k\right)}=\sqrt{Var(x^{\left(k\right)})}$$

The Reduction block is inserted between two Inception-ResNet modules, which is primarily used to adjust the width and depth of the network, serving as a pooling function. This module also employs multi-scale convolutional kernel and pooling with a parallel structure to mitigate model overfitting.

Next, a down-sampling operation is performed on the overall feature information at the Average Pooling layer to reduce the parameter dimensions and allow more complete information to be passed on. To prevent overfitting of the model, some neurons are removed randomly during each training iteration through the Dropout layer, and the outputs of these neurons will not be updated in this training. This reduces the overall complexity and number of parameters of the neural network, which enhances the generalization ability of the model. In this way, the Dropout layer helps the model learn more robust features and decreases the interdependencies among neurons, enabling the model to perform better on different data distributions. Finally, the fully connected layer and the SoftMax function are used to output the recognition results.

The model collected a total of 4752 eye diagrams of the 100 Gbps Apol-CRZ-FSK, CRZ-FSK, and DQPSK modulation formats at different OSNR levels and distances, respectively. The entire image dataset was randomly divided into a training set (80%) and a test set (20%), which means 3802 images were used for training and 950 images for testing, thereby facilitating the research on modulation format recognition.

### 3.2. Results and Discussion

#### 3.2.1. Modulation Format Identification Results

**Figure 9:**
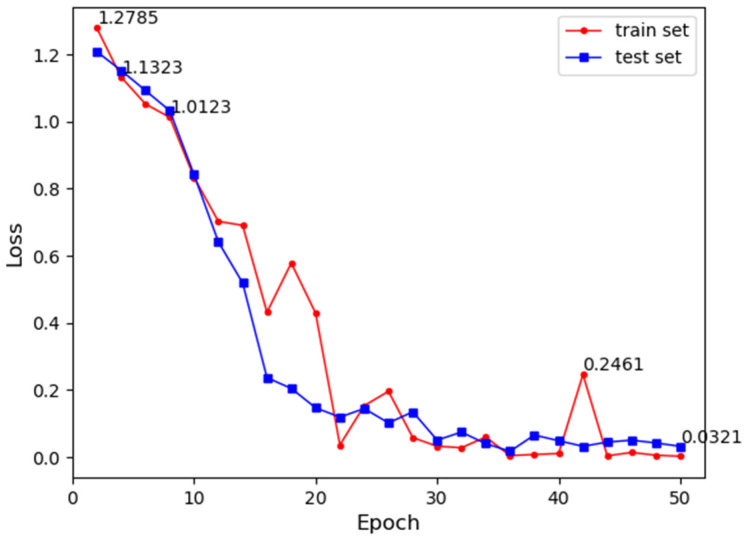
Loss values for training and test sets.

The test results of the loss values for the training and test sets are shown in Figure 9, where the loss function is used to evaluate the convergence of the model. It can be observed that the loss values for both the training and test sets significantly decrease with the increase in the number of training epochs, indicating a very stable convergence of the model. After 50 epochs, the loss values for the training and test sets are 0.0713 and 0.101, respectively. The low loss values suggest that the model has good robustness.

**Figure 10:**
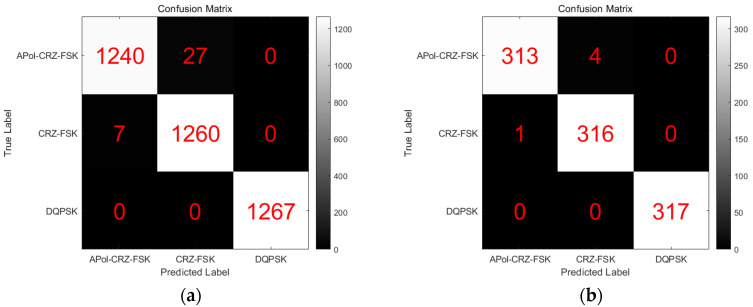
MFI confusion matrix for training and testing sets: (**a**) training set output confusion matrix; (**b**) testing set output confusion matrix.

The MFI for the training and test sets is depicted in Figure 10. Each column of the matrix signifies the actual output types of modulation formats, while each row represents the target types of modulation formats. By examining the data between the rows and columns, one can discern the number of instances correctly and incorrectly identified. There are 1267 training images and 317 test images for each modulation format. As shown in Figure 10a, only the DQPSK format achieves a 100% MFI classification accuracy, while the MFI classification accuracy for the Apol-CRZ-FSK and CRZ-FSK formats can reach up to 99%. Figure 10b indicates that only the DQPSK format can attain a 100% MFI classification accuracy. In contrast, misclassification occurs for the modulation formats Apol-CRZ-FSK and CRZ-FSK. For example, for the 317 images using Apol-CRZ-FSK, four instances were misclassified as CRZ-FSK. The classification accuracies of Apol-CRZ-FSK and CRZ-FSK were 98.73% and 99.68%, respectively, and their MFI errors were because the format of Apol-CRZ-FSK is an orthogonal polarization based on CRZ-FSK, and both formats share similar characteristics. However, the probability of recognition error is extremely small, and excellent performance has been achieved in the recognition of three optical signal formats.

#### 3.2.2. Effect of Different Factors on MFI

**Figure 11:**
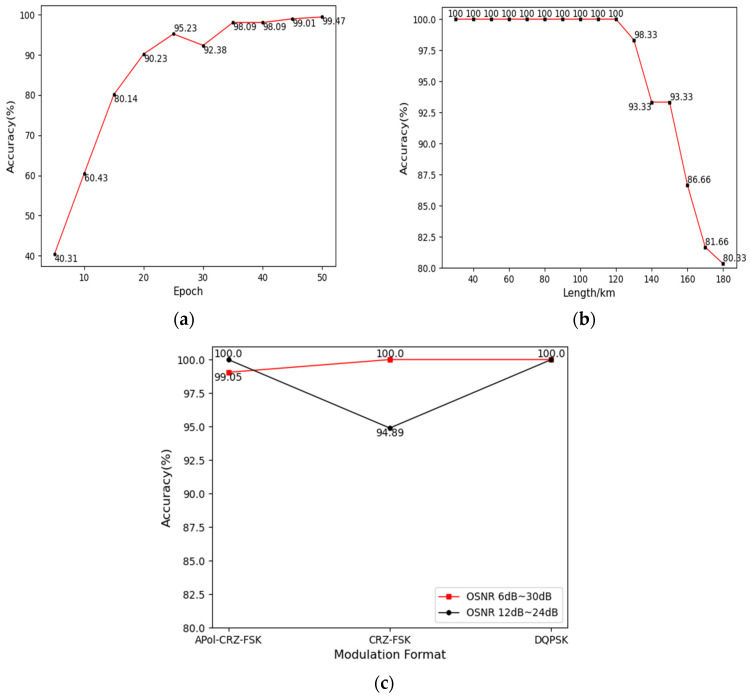
Effect of different factors on model MFI: (**a**) accuracy of the model at different number of rounds; (**b**) effect of different transmission distances on MFI; (**c**) effect of different signal-to-noise ratios on MFI.

The model’s performance under different epochs, transmission distances, and OSNR conditions are studied in this paper. The accuracy of the model under various epochs is shown in Figure 11a. When the number of epochs is too small, the effective features within the dataset are not fully extracted, or the model’s parameters are not sufficiently adjusted during the training phase, hence a low accuracy achieved within a short period. The overall trend is an increase in accuracy with the number of epochs, as the model parameters are gradually optimized and more features are extracted, leading to a step-by-step improvement in performance.

The accuracy reaches 99% at 50 epochs. Further increasing the number of epochs, the performance does not improve further but rather fluctuates around a certain value, due to the model reaching saturation in feature extraction. The MFI accuracy has reached a satisfactory level within the tested range of epochs, proving that the model has strong robustness under the factor of epochs.

With an increment of 10 km for each step, the transmission distances of the three types of signals vary from 30 km to 180 km. The impact of different transmission distances on the model’s MFI is shown in Figure 11b. It can be observed that when the transmission distance is less than or equal to 120 km, the MFI accuracy of the model is unaffected by the distance and remains consistently at 100%. However, at a transmission distance of 130 km, the accuracy begins to decline; the longer the signal transmission distance, the more severe the signal degradation, the higher BER, and the more pronounced the eye diagram distortion. The damage to the signal due to transmission distance will affect the model’s modulation format recognition to a certain extent. As the transmission distance continues to increase, the features of the eye diagram will gradually become blurred, and partial or local distortion may occur.

Within the test range from 130 km to 180 km, the accuracy of the model can be maintained at more than 80.33%. The dispersion, nonlinear effect, and other fiber transmission characteristics will affect the speed and quality of the signal transmission, which can be compensated by the corresponding digital signal processing algorithms to ensure the integrity and quality of the signal.

Finally, the effect of different OSNRs on the model MFI was investigated. During the dataset acquisition, the OSNR was varied from 6 dB to 30 dB in steps of 2 dB. The recognition accuracy of the Inception-ResNet-v2 model is shown in Figure 11c. The accuracy of the model can be maintained at 99.68% when the OSNR is varied from 6 dB to 30 dB, and the accuracy of the model can be maintained at 98.29% when the OSNR is varied from 12 dB to 24 dB, and the overall effect is as expected.

#### 3.2.3. Comparison with Different Methods

**Figure 12:**
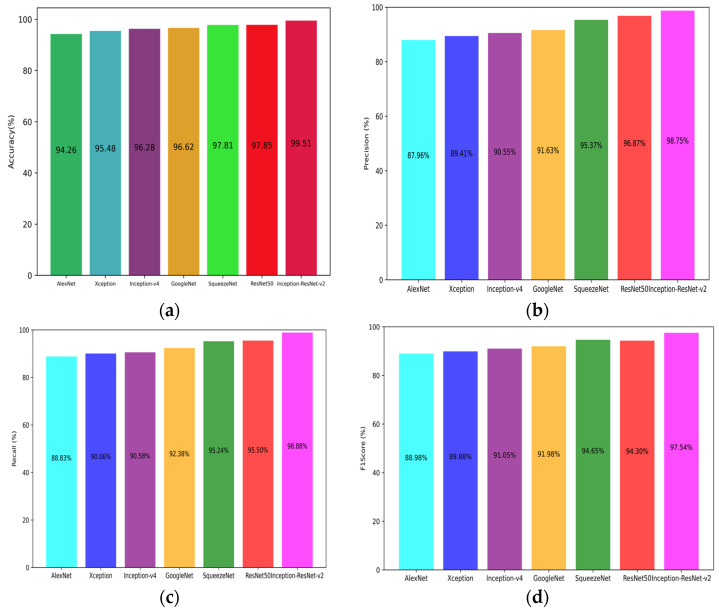
Comparative analysis of different modulation format recognition methods: (**a**) accuracy; (**b**) precision; (**c**) recall; (**d**) F1 score.

To further validate the performance of the Inception-ResNet-v2 model, it was compared with a total of six deep learning methods—AlexNet, ResNet50, GoogleNet, SqueezeNet, Inception-v4, and Xception—with the same simulation conditions, and the comparison results are shown in Figure 12, the different colors in the figure are used to distinguish between six different deep learning methods, with the specific method names indicated on the horizontal axis. The selected six deep learning methods are highly regarded and widely adopted in the field of image recognition and classification. They represent neural network architectures from different developmental stages and with different design philosophies, each having unique characteristics and applicability to various scenarios; researchers often use these methods for modulation format identification [15,16,17,18,19,20], which are representative to some extent. To ensure fairness and accuracy in the comparison, all participating methods utilized a unified dataset. This dataset comprehensively covers optical signals under multiple modulation formats and extensively includes various optical signal-to-noise ratio (OSNR) ranges and transmission distances, aiming to simulate the complex and varied actual operating environments in optical communication systems. To objectively assess the generalization ability of each model, a random split strategy was adopted to divide the dataset into two parts, forming a training set and a test set with independent splits each time, and the average accuracy was calculated to mitigate the potential interference of randomness on the experimental results.

As shown in Figure 12a, the accuracy of all six neural network architectures exceeded 95%, indicating a strong recognition capability across these models. Notably, the accuracy of the SqueezeNet model reached 97.89%, while the accuracy of the Inception-ResNet-v2 model was even higher at 99.47%, representing an improvement of 1.58% over the SqueezeNet model, which highlights the significant advantage of the Inception-ResNet-v2 model in terms of accuracy.

From Figure 12b, the precision of the Inception-ResNet-v2 model reached 98.75%, which is 10.79% higher than the lowest precision of the AlexNet model. High precision means that when the model predicts a sample to belong to a certain category, the probability of this prediction being correct is high, thus the Inception-ResNet-v2 model performs excellently in reducing false positives and has high reliability.

Figure 12c shows that the recall rate of the Inception-ResNet-v2 model was 98.88%, meaning that it effectively identified almost all samples that belonged to the positive class (i.e., specific modulation formats). A high recall rate is crucial for avoiding missed detections, especially in application scenarios requiring comprehensive detection of specific modulation formats.

Finally, Figure 12d reveals that the F1 score of the Inception-ResNet-v2 model was 97.54%, which is a composite evaluation metric combining precision and recall. The high F1 score further demonstrates that the Inception-ResNet-v2 model not only maintains high precision but also sustains a high level of recall, thereby showcasing more balanced and efficient recognition capabilities in practical applications.

## 4. Conclusions

This paper proposes and studies an advanced modulation format Apol-CRZ-FSK with high nonlinear resistance, to achieve a solution for 4 × 100 Gbps computing optical networks. Simulation results indicate that when the input fiber power is low and the impact of nonlinear effects is negligible, the Apol-CRZ-FSK modulation format does not demonstrate significant performance advantages in the 4 × 100 Gbps system. However, as the optical power gradually increases and the transmission distance extends, the Apol-CRZ-FSK signal exhibits better nonlinear resistance when nonlinear effects become the primary factor affecting system performance. The longest single-span transmission distance of the Apol-CRZ-FSK signal is 16 km longer than that of the CRZ-FSK and 23 km longer than that of the DQPSK modulation formats. At a transmission distance of 120 km, when the launch fiber power is 20 dBm, the Q-factors of the four channels for the 4 × 100 Gbps Apol-CRZ-FSK signal are 2.9 dB, 0.8 dB, 4.5 dB, and 2.5 dB higher than those of the CRZ-FSK signal, respectively; and 8.8 dB, 6.9 dB, 9.4 dB, and 8.2 dB higher than those of the DQPSK signal, respectively. Under multi-span transmission conditions, the furthest transmission distance of the 4 × 100 Gbps Apol-CRZ-FSK modulation signal is 980 km and 480 km farther than that of the CRZ-FSK and DQPSK modulation signal, respectively. In summary, the Apol-CRZ-FSK modulation signal has certain transmission performance advantages. The demodulation on the receiving side of the Apol-CRZ-FSK signal does not require complex coherent demodulation, effectively reducing system costs and saving energy with direct detection. Furthermore, in response to the transmission demands and signal perception recognition requirements in future optical networks, this study identifies three different modulation formats by the Inception-ResNet-v2 neural network model. Simulation results show that the Inception-ResNet-v2 model achieves the highest performance compared to other six methods, providing an effective solution for the related research and development of optical networks.

## 5. Patents

Chinese National Invention Patent, An Optical Signal Modulation Scheme and Its Transmission System, 2023.

## Data Availability

Data will be provided on suitable request.
